# Mental health in Malawi

**DOI:** 10.1192/bji.2019.28

**Published:** 2020-05

**Authors:** Philippa Lilford

**Affiliations:** ST5 Psychiatry, Severn Deanery. Email: p.lilford@nhs.net

**Keywords:** Low and middle income countries, transcultural psychiatry, psychotic disorders

## Abstract

This is a reflective essay about the time I spent volunteering in mental healthcare in Malawi, prior to commencing my psychiatry training. The burden of illness I saw was enormous and the resources very limited; however, I describe some particular experiences where we were able to deliver excellent care, and which made me reflect about mental health services in low-income countries in general. Details of the patients discussed in this essay have been fully anonymised.

A few years ago, before starting my psychiatry training, I travelled to Malawi to volunteer in psychiatry for 3 months. On the way there, feeling that I was at the start of a great adventure, I started reading the 2007 *Lancet* Series on Global Mental Health.^1^ This would be the first time since medical school that I had undertaken a post in psychiatry. I had visited Africa numerous times before and experienced medicine in South Africa during my elective, but I felt nervous at the challenge that lay ahead. Malawi is one of the poorest countries in the world and, with a population of 16 million, had just two qualified psychiatrists; neither doctor was Malawian.

I was based in the out-patient department of the Queen Elizabeth Hospital in Blantyre. This was a walk-in clinic which was officially held in the morning, 3 days a week. In reality, it lasted most of the day from Monday to Friday. The clinic was staffed by three psychiatric nurses, in addition to the two permanent consultant psychiatrists. They were supplemented by variable numbers of elective students and consultant psychiatrists who were volunteering with the Scotland Malawi Mental Health Education Project. Each day the corridor leading to the clinic would overflow with patients and their guardians. Guardians are family members that have taken on the task of looking after a patient when they are unwell. I had imagined that stigma would be rife, and that patients would have been ostracised and left to attend clinic alone. To my surprise, the majority of patients attended with a guardian who provided a collateral history, ensured patients took their medication and provided a safety net that allowed patients to be sent home. If patients were admitted to the Queen Elizabeth hospital, a guardian remained with them on the ward. The guardian would sleep on the floor or outside, and they would cook for and clean the patients for the duration of their admission.

Around 40 patients would arrive at the clinic daily, many walking for hours from distant villages. There is very little public transport in Malawi, and patients from further afield would come by small private vans. The clinic and medication are free and are funded by the government or by private donors. However, transport costs are borne by the patient and their families, and those costs often meant that patients could not return as often as the service required. Most patients could speak only the Malawian language, Chichewa, and so it was necessary to obtain their complex psychiatric stories with the assistance of a translator.

The nurses had many skills. For example, they would administer intravenous diazepam to very agitated patients who had been brought in by the police, and the next day I would see them conducting a session on psychoeducation to improve medication adherence. The available medication could vary from week to week, depending on governmental supply and private donations. Some weeks, we would have a range of atypical antipsychotics, selective serotonin reuptake inhibitors and tricyclic antidepressants. The supply of diazepam and chlorpromazine fortunately remained abundant. A small proportion of patients could afford to pay for their medication, in which case a prescription could be written for an alternative medication supplier by a private pharmacist close to the hospital.

In my first week, I received a referral from my friend who was working as a registrar on the medical ward in the Queen Elizabeth hospital. I went to see the patient with one of the psychiatric nurses. We turned the corner into the heaving ward and easily identified our patient. A mattress had been placed on the floor in the corridor, and his arms and legs were tied together with rope. He was spitting and shouting ‘fire’ repeatedly. When I walked towards him, he started rolling in my direction trying to bite me. He had been an in-patient for a week, but he had been placed in the corridor between two bays where neither medical team was required to take ownership of him. He had not been referred to psychiatric services, nor had he been given benzodiazepines. He had not been given any food and had barely drunk any liquid. No physical observations had been performed or any blood drawn.

His father told us he had become acutely agitated over a few days. He had stopped making sense and started talking about religious topics. He did not have a significant history of alcohol intoxication. His father did not think he took drugs and there was no mental illness in his family. His blood test results were unremarkable, and his HIV status was negative. We started him on regular diazepam and chlorpromazine. He became calm and compliant and he was discharged on chlorpromazine.

I saw him in clinic a week later. He had clear psychotic features and no side-effects from the medication. I increased his dose of antipsychotic medication and saw him again 2 weeks later. He remained floridly psychotic and he had attempted to hang himself. I spoke with my consultant and we agreed that he required admission under the Mental Health Act. There are many criticisms that can be made against the Malawian version of the Mental Health Act, for example, the length of detention can go unchallenged, but at least a legal framework to detain people against their will does exist.

He was admitted to Zomba Mental Health Hospital, the only state-funded in-patient psychiatric hospital in Malawi, which is situated about an hour's drive from Blantyre. Zomba Mental Health Hospital is run by clinical officers who have studied for 3 years and then undertaken a Bachelor of Sciences in Mental Health. I spent a week in that psychiatric hospital teaching medical students how to take psychiatric histories and perform mental state exams. There are only 250, which must be reserved for the most seriously unwell patients. Interestingly, another common reason for detention arises when a patient's mental health is interfering with their physical health. For example, many patients are admitted so that regular antipsychotic therapy can be administered in order to increase adherence to their anti-tuberculosis and antiretroviral medication.

Most patients on the ward were naked, and they were often showered outside with a hose. A very large number had notable extra-pyramidal side-effects from their medication. The nurses based on the ward were remarkably integrated with the patients. I went onto one ward with a group of students and saw a nurse playing football with a patient. I returned 4 hours later, and they were still kicking the ball around, having recruited more patients into their game. There was a disco held every week, and there was no obvious division between staff and patients, who danced together for hours.

Often, our psychiatric service was the last option for families who had exhausted their use of faith healers. I saw one lady who had developed postnatal psychosis 2 years previously. I started her on a low dose of chlorpromazine and her symptoms quickly resolved. Her older children were delighted that their mother had recovered from her mental illness. I wondered what effect this remarkable improvement in her condition would have on her husband, who had been the driving force behind her previous consultations with traditional healers. He too was struck by the difference, but he did not feel ashamed about her disease, nor did he express any reservations about their attendance at our clinic; he was only remorseful that he had not brought her earlier. This made me reflect on the role of traditional healers. It was not financial expediency that had driven the family to seek the traditional route, since healers charge for their services and the clinic did not charge patients. There is much debate in low- and middle-income countries where traditional healers are still practising widely about whether traditional medicine could act as an access point to mainstream medical facilities, and whether their liaison with mainstream medicine could be enhanced.^2^

On my journey, while I was reading the *Lancet* Series, I thought I would be left feeling hopeless and frustrated at the lack of care I would be able to offer. Of course, many of the features of care in societies that lack modern medical facilities, which were highlighted in the series, did resonate with my experience. The burden of mental illness, both on the country and on individuals, was every bit as great as I had anticipated. Resources are incredibly scarce and mental health is not a prioritised service in Malawi, or in most other countries in sub-Saharan Africa. Despite this reality, I am left with a feeling that is there is still promise for the future. The interventions that I witnessed do work so effectively in the clinic I visited, and they are low cost. The psychological and pharmacological therapies that were delivered need to be scaled up and taken out of the clinic and into the community. Perhaps liaison with traditional healers could have a role in this extension of services. The social structure I observed, where patients were ‘allocated’ a family member to care for them in times of illness, is an immensely positive and useful resource for therapy and rehabilitation in terms of mental healthcare. I am delighted to report that there are now two recently qualified Malawian doctors who are working as consultant psychiatrists in that country. Exploring how people with mental health problems present in Malawi and the role of traditional healers continues to be an interest of mine, and I hope to return at some point during my career and explore this further.
